# Putrescine: A Key Metabolite Involved in Plant Development, Tolerance and Resistance Responses to Stress

**DOI:** 10.3390/ijms23062971

**Published:** 2022-03-10

**Authors:** Ana Isabel González-Hernández, Loredana Scalschi, Begonya Vicedo, Emilio Luis Marcos-Barbero, Rosa Morcuende, Gemma Camañes

**Affiliations:** 1 Institute of Natural Resources and Agrobiology of Salamanca (IRNASA), Consejo Superior de Investigaciones Científicas (CSIC), 37008 Salamanca, Spain; emiliol.marcos@irnasa.csic.es (E.L.M.-B.); rosa.morcuende@irnasa.csic.es (R.M.); 2 Grupo de Bioquímica y Biotecnología, Departamento de Biología, Bioquímica y Ciencias Naturales, Universitat Jaume I de Castellón, 12071 Castellón de la Plana, Spain; scalschi@uji.es (L.S.); bvicedo@uji.es (B.V.); camanes@uji.es (G.C.)

**Keywords:** putrescine, plant growth, abiotic stress, biotic stress

## Abstract

Putrescine (Put) is the starting point of the polyamines (PAs) pathway and the most common PA in higher plants. It is synthesized by two main pathways (from ornithine and arginine), but recently a third pathway from citrulline was reported in sesame plants. There is strong evidence that Put may play a crucial role not only in plant growth and development but also in the tolerance responses to the major stresses affecting crop production. The main strategies to investigate the involvement of PA in plant systems are based on the application of competitive inhibitors, exogenous PAs treatments, and the most efficient approaches based on mutant and transgenic plants. Thus, in this article, the recent advances in understanding the role of this metabolite in plant growth promotion and protection against abiotic and biotic stresses will be discussed to provide an overview for future research.

## 1. Introduction

**Figure 1:**
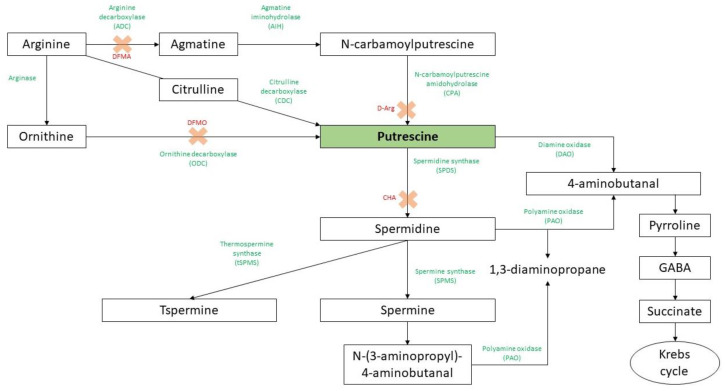
Putrescine biosynthesis and catabolism in plants. The green words indicate the enzyme activities, and the red ones followed by a cross are referred to competitive enzyme inhibitors (DFMA: difluoromethylarginine; DFMO: difluoromethylornithine; D-Arg: D-Arginine; CHA: cyclohexylamine). Adapted from Wojtasik et al. [20] and Chen et al. [4].

Polyamines (PAs) are small, low molecular weight, and ubiquitous polycations found in eukaryotic and prokaryotic cells [1,2]. In higher plants, they could be found not only in the free form but also as conjugates bound to phenolic acids (hydroxycinnamic, coumaric, caffeic, or ferulic acid) or to biomacromolecules such as proteins and nucleic acids in order to regulate the free PAs intracellular levels or control enzyme activity, DNA replication, gene transcription, cell division, and membrane stability [3,4]. The most common PAs in higher plants are diamine putrescine (Put), triamine spermidine (Spd), tetramine spermine (Spm), thermospermine (Tspm), and cadaverine (Cad) [5,6,7,8] (Figure 1). Among them, Put is the central product of the PA biosynthetic pathway and the most abundant PA in nature, being mainly synthesized by two pathways derived from ornithine (Orn) or from arginine (Arg) as a result of the activity of ornithine decarboxylase (ODC, EC 4.1.1.17) or arginine decarboxylase (ADC, EC 4.1.1.19), respectively [9]. Orn is produced from Arg by arginase, and then ODC eliminates a carboxyl group of Orn to generate Put and CO_2_ [10]. The Arg pathway includes the following steps: (i) arginine decarboxylation to agmatine catalyzed by ADC; (ii) deimination of agmatine by agmatine iminohydrolase (AIH, EC 3.5.3.12) to form N-carbamoylputrescine (NCP) and NH_3_, and (iii) hydrolysis of NCP to Put by N-carbamoylputrescine amidohydrolase (CPA, EC 3.5.1.53), releasing NH_3_ and CO_2_ [11]. It should be noted that the *ODC* gene is not present in *Arabidopsis thaliana* and other plants of the Brassicaceae family [12]. In *A. thaliana*, two genes encoding ADC, *ADC1* and *ADC2*, were identified and are expressed in a tissue-specific manner [13]. This gene duplication seems to be related to the differential regulation of gene responsiveness [14]. A third pathway uncovered to date only in sesame involves the conversion of Arg to citrulline (Cit) and subsequent decarboxylation catalyzed by citrulline decarboxylase (CDC) to generate Put [4]. Once Put is formed, the Spd and Spm are synthesized from Put and aminopropyl residues by the activity of Spd synthase (SPDS) and Spm synthase (SPMS), respectively [15], whereas the Tspm is then produced through the isomerization of the Spm by the enzyme thermospermine synthase (tSPMS) called ACAULIS5 (ACL5) [16,17]. The breakdown of PAs is mediated by amine oxidases, including the diamine oxidase (DAO) and polyamine oxidase (PAO) [4,18,19].

The activity of ADC and ODC can be inhibited by the irreversible competitive inhibitors difluoromethylarginine (DFMA) and difluoromethylornithine (DFMO), respectively [21,22], as well as by the reversible inhibitor of ADC, D-Arginine (D-Arg) [23] (Figure 1). Intriguingly, plants treated with biosynthetic inhibitors displayed a reduction in stress tolerance, which was reversed when PAs were applied exogenously [24]. However, the use of these chemical inhibitors seems to be limited due to their stability and specificity, and it is influenced by the concentration, the plant system, and the induction of compensatory mechanisms [25]. Therefore, the use of most sustainable approaches such as mutants or transgenic plants will allow a deeper understanding of the direct implication of PAs in plant systems [26].

PAs play a role in physiological processes such as embryogenic competence, root growth, organogenesis, flower development, fruit ripening, or programmed cell death [27,28], as well as plant defense responses [29,30]. Their cationic nature explains most of their biological activity. However, the numerous biological interactions where PAs are involved makes it difficult to determine their role in plant growth and development. Put is not only a signal molecule by itself, but also interacts with numerous molecules such as phytohormones and gas molecules, among others. Put mostly displayed the opposite effects of Spd and Spm, which suggests that each PA may play a distinctive role in plant metabolome and transcriptome, as it was reviewed by Anwar et al. [31]. Generally, Put was positively linked with the gene expression for abscisic acid (ABA) biosynthesis and indole acetic acid (IAA) and salicylic acid (SA) levels, albeit downregulating those of ethylene, jasmonates (JA), and gibberellin (GA) biosynthesis. Moreover, Put seems to play a neutral to positive role in regulating the JA or brassinosteroids signaling pathways [31]. However, Put does not appear to affect cytokinin (CK) biosynthesis or signaling. Likewise, ABA seems to crosstalk with PAs in regulating abiotic stress responses, including reactive oxygen species (ROS), nitric oxide (NO), and changing ion homeostasis [31,32]. NO production could be mediated by H_2_O_2_ as the result of PAs oxidation by DAO and PAO or by other unknown mechanisms that could be related to the PAs pathway [33]. The positive regulatory role of NO in the increase of the expression of genes involved in PA biosynthesis and the decrease of PAO enzyme activity was previously reviewed, confirming the role of NO in PA homeostasis [34]. It should also be noted that H_2_S, an endogenous gas transmitter, could also play a negative and positive role in plants, acting as a toxic intermediate of cellular metabolism or as a signaling molecule, respectively [35,36]. Its role in seed germination, adventitious rooting, senescence, and protection against abiotic stresses was previously reported [37]. Thus, it could be speculated that both gas molecules could behave as a link messenger in stress responses mediated by PAs, filling a gap between many known physiological effects of Put and stress tolerance.

In addition, plants are often subjected to various abiotic and biotic constraints that limit plant growth and productivity. PAs are one of the involved pathways protecting plants, even though their catabolic products could damage plants [38]. Nonetheless, the regulation of plant growth and stress responses by PAs is not fully understood. Thus, the main goal of this review is to present an overview of the recent research about the role of Put in plant growth and development, tolerance, and resistance against the major abiotic and biotic stresses to provide a basis for future research on Put action in plant systems.

## 2. Plant Growth Responses

PAs are considered a class of plant growth regulators [39]. In general, enhanced plant growth and metabolism are associated with greater PA biosynthesis and higher PA content [40,41]. PAs show specific tissue and organ distribution and different localization patterns within cells, which are related to their unique functions. Among the main PAs, Put is the most abundant in leaves, and it is found to accumulate in the cytoplasm [40]. It is also demonstrated that the chemical or genetic depletion of Put is lethal for many organisms, not only for plants, suggesting that Put may play an essential role in growth and development [42,43]. However, the molecular mechanisms behind these roles remain ambiguous. It was suggested that the enhancement of plant growth might be due to the fact that PAs act as hormonal second-messengers of cell proliferation and differentiation in many processes or regulate plant sensitivity to auxins/CKs ratio. In addition, the metabolism of PAs was related to the production of NO, which is considered an essential signaling component for plant growth [44].

In this regard, many studies showed that modifications in Put content can affect root growth and development. For example, the depletion of Put due to a decrease of ADC activity led to a reduction of root length in *Phaseolus vulgaris* plants [45]. Moreover, Lee [46] showed that Put treatment in concentrations varying from 0.01 to 1 mM enhanced root elongation in the excised root of *Oryza sativa* grown under in vitro conditions at 25 °C. Likewise, DFMO was found to inhibit root elongation and PAs levels in roots, and these effects were reversed by DFMO plus Put co-treatment, or 1 mM Put exogenous treatment. Similarly, Tarenghi et al. [47] showed that 1 mM Put exogenous treatment led to an increase of Put level in roots and to an increase of root length in strawberry microcuttings. In the same way, Wu et al. [48], when investigating the effect of an arbuscular mycorrhizal fungus (AMF) and Put on root development, plant growth, and biomass production of 4 months-old trifoliate orange, observed that total root length, projected area, surface area, and root volume were significantly increased by the Put treatment compared to the sole AMF treatment. However, DFMO treatment was found to cause an increase in root system length in parallel with a decrease in Put levels in excised roots from plants of *Nicotiana tabacum* [49]. These changes were reversed when 1 mM Put was added to the DFMO treatment. In accordance with previous observations, in *Pringlea antiscorbutica*, a decrease of the Put pool seemed to enhance primary root growth in a concentration-dependent manner. Similar results were obtained by Tang et al. [50] in Virginia pine plantlets. They demonstrated that Put application at 0.001 mM improved rooting frequency and promoted root elongation while Put treatment at 0.01–1 mM decreased rooting frequency and reduced root elongation. Likewise, simultaneous silencing of the two *ADC* genes in *A. thaliana* led to a significant reduction in primary root length [51]. Put also plays a crucial role in rooting of *Decalepis hamiltonii* since it is shown that supplementing Put in the rooting medium enhances the quantity and quality of roots [52]. Moreover, spraying *Antirrhinum majus* with 200 mg L^−1^ of Put has a significant effect on root length and fresh (FW) and dry weight (DW) [53]. Studies on Basil plants revealed that the application of Put, Spd, or Spm at different concentrations increased root FW and DW when compared with the control plants [54]. Furthermore, Hashem et al. [23] progressively reduced Put biosynthesis by inhibiting ADC1/2 enzyme activity using the competitive inhibitor D-Arg, leading to increased root growth at low D-Arg concentrations and progressively decreased root growth at higher ones. A similar trend was also observed for the meristematic zone size. They next investigated whether reduced Put affects auxin and CK signaling since both hormones are involved in the regulation of root meristem size. Auxin signaling displayed a U-shaped trend as D-Arg increased, whereas CK progressively decreased with increasing concentrations of the inhibitor. Taken together, all of these results highlight the fact that there are important inter-species differences, which might also depend on culture conditions.

The root system plays an important role in water and nutrient uptake. It was observed that nitrogen sources such as ammonium (NH_4_^+^) and nitrate (NO_3_^−^) impact differently on some physiological and biochemical processes in higher plants. In fact, detailed analysis from Houdusse et al. [55] revealed that the foliar free Put content was well correlated with the intensity of the negative effects of NH_4_^+^ as the sole N source on the development of wheat (*Triticum aestivum*) and pepper (*Capsicum annuum*) plants. In the same study, it was observed that plants supplied with NH_4_NO_3_ exhibited a decline in free Put content when compared with those fed with NH_4_^+^ alone, in both roots and leaves of wheat and pepper plants. Moreover, the effect of different concentrations of Put on the activity of enzymes of N assimilation was examined in maize seedlings. It was observed that both glutamate dehydrogenase (GDH) and glutamine synthetase (GS) activities were enhanced at low concentrations of Put while glutamate synthase (GOGAT) activity increased with increasing Put concentration [56]. In a recent study, González-Hernández et al. [57] tested the effect of defective *ADC* and *ODC* gene expression on the root architecture development of tomato plants (*Solanum lycopersicum*) under both NO_3_^−^ and NH_4_^+^ nutrition. The *ADC* transgenic silenced tomato seedlings showed an increase in FW, shoot length, lateral root number and shoot:root ratio under NO_3_^−^ supply and an enhancement in FW, and shoot and root length under NH_4_^+^ supply. However, *ODC* transgenic silenced tomato seedlings displayed greater weight and shoot length with NO_3_^−^, whereas a decrease in lateral root density was found with NH_4_^+^.

Put also modulates shoot growth. Related to this, Nahed and Lobna [58] showed that foliar application of Put significantly increased plant height, number of leaves per plant, and FW and DW of leaves per plant when compared with untreated plants. Similarly, Youssef et al. [59] reported that foliar application of Put to *Matthiola incana* plants significantly promoted plant height, the number of leaves per plant, and FW and DW of leaves per plant at the vegetative growth stage. In periwinkle plants (*Catharanthus roseus*), Talaat et al. [60] observed that foliar application of Put had beneficial effects on different growth parameters of shoots and leaves at successive developmental stages, which were correlated with an increase in the endogenous GA3, IAA, CKs, and ABA levels. Moreover, leaf spraying with Put and thiamine showed that Put treatment significantly increased plant height, number of shoots, number of leaves, leaf, stem FW and DW, and stem diameter in *Dahlia pinnata* plants [61]. In *Antirrhinum majus*, foliar Put spray also led to an increase in growth parameters such as plant height, number of shoots, number of leaves, leaf area, and stem FW and DW [53]. In a study on the effect of humic acid and Put on rose, it was found that humic acid with Put treatment increased stem FW and DW, leaf area, and plant height [62]. Tomato seedlings exposed to cinnamic acid decreased root and shoot lengths and FW and DW. These parameters increased when Put was applied in combination with cinnamic acid or alone [63]. A study on cucumber reported that root application of humic acid caused a significant increase in shoot growth that was associated with an enhancement in the shoot concentration of several CKs and PAs (principally Put), concomitant with a decrease in roots [64]. In addition, Krizek et al. [65] showed that the treatment of cucumber plants with Put increased the DW of shoots and leaf area but had no effect on root DW. *Oryza sativa* seed treatments with Put resulted in earlier and enhanced germination along with improved shoot and root lengths and seedling FW and DW [66].

In safflower plants subjected to different Put concentrations, it was found that the maximum shoot regeneration occurred with the highest concentration of Put tested, which also had the highest peroxidase activity [67]. Moreover, the addition of Put (40 mM) resulted in increased shoot proliferation, in vitro flowering, and increased endogenous levels of PAs in *Cichorium intybus* cv. Lucknow [68].

Several studies with different plant species provided evidence of the effect of Put on photosynthetic pigments. Indeed, Put application increased the content of chlorophyll in several ornamental plants [58,69,70], while in *Salvia splendens* it not only increased the chlorophyll content but also the anthocyanin and soluble sugars [71]. Total photosynthetic pigments in fresh leaves were significantly promoted because of the application of Put in chickpea plants (*Cicer arietinum*) [72]. Moreover, tomato seedlings showed higher levels of photosynthetic pigments, protein and sugar, and higher nitrate reductase activity when Put was exogenously applied [63]. It was also reported that foliar application increased the content of chlorophylls (a and b) as well as carotenoids in a concentration-dependent manner in periwinkle plants. These results resemble those reported in wheat after application of either Arg or Put [73]. In a recent study carried out with basil plants (*Ocimum basilicum*), Danaee and Abdossi [54] showed that the total leaf chlorophyll and vitamin C contents were increased in plants treated with Put at 100 ppm compared to untreated ones. Moreover, El-Bassiuony and Bekheta [74] reported an accumulation of total carbohydrates content in wheat plants treated with Put, suggesting the stimulation of the photosynthetic assimilation of CO_2_. Regulation of ATP production is one of the most important processes for a photosynthetic organism which determines the amount of energy available for energy-consuming processes. Related to this, Ioannidis et al. [75] revealed that Put is an efficient stimulator of ATP synthesis by demonstrating that Put can increase light energy utilization through stimulation of photophosphorylation.

Put may influence the different stages of plant development, including flowering. In this context, Singh and Bala [76] observed that an increase in Put concentration delayed the bud formation in chrysanthemum cv. ‘Punjab Shyamli’. On the contrary, studies in *Dendrobium nobile* plants with higher levels of Put and Spd in the leaves showed that these plants had more flower buds, and they also presented not only more flowers but also with a larger average flower diameters [77]. Similarly, the application of 200 ppm of Put increased the number of flowers and their FW and DW in *Dianthus caryophyllus* and *Gladiolus grandflorum* [58,78]. *Antirrhinum majus* plants treated with different concentrations of Put significantly increased the number of inflorescences per plant, yield of spike, and FW and DW of inflorescences per plant compared with untreated plants [79]. Treated plants also showed a decrease in the number of days for flowering. In addition, treatment with Put induced flowering in chicory shoot cultures [68]. However, in *Fragaria × ananassa* Duch. cv. Selva, *Calendula officinalis,* and *Rosa hybrida* cv. ‘Herbert Stevens’ plants, no significant effect of Put treatment on the number of flowers was found [70,80,81]. In other studies, foliar application of Put with α-tocopherol enhanced the anthocyanin content in the inflorescences and the total carbohydrates in shoots and inflorescences of *A. majus* [53].

Put appears to be involved in a wide range of physiological processes in citrus plants which were recently reviewed by Killiny and Nehela [82]. In summary, Put positively regulated root and shoot growth, increased the number of total flowers per tree, fruit yield per tree, fruit weight, and fruit diameter. Likewise, exogenous Put not only significantly increased chlorophyll a, total carotenoid and total chlorophyll contents, but also the photosynthetic rates.

**Figure 2:**
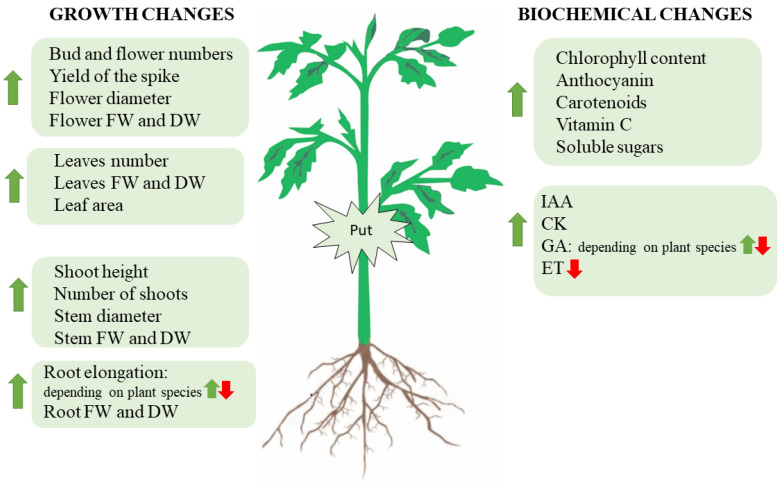
Developmental and biochemical changes produced in plants under Put treatment. FW: fresh weight; DW: dry weight; IAA: indole acetic acid; GA: gibberellins; CK: cytokinins; ET: ethylene.

Interestingly, it has been shown that plant hormones involved in plant growth and development are associated with the metabolism of PAs. Related to this, El-Bassiouny [73] and Bekheta and El-Bassiouny [83] demonstrated that exogenous application of Put on *Pisum sativum* and wheat increased IAA, GA, and CK levels and decreased ABA content, respectively. Moreover, constitutive overexpression of *ADC2* (*35S:AtADC2*) in *A. thaliana* led to a more than 16-fold increase in the levels of Put in the transgenic plants when compared to the wild type, whereas the level of Spd and Spm remained unchanged [84]. These transgenic plants were dwarfed with delayed flowering. Transcriptomic and metabolomic analysis revealed that accumulation of Put downregulated the expression of dioxygenase genes (*GA20ox1*, *GA3ox1*, and *GA3ox3*), which are involved in the last step of GA metabolism and decreased the content of bioactive GA4 and GA1, and of GA9 (a precursor of GA4). No changes in the expression of genes encoding earlier enzymes in the GA biosynthesis pathway were detected by microarray analysis. Furthermore, *ADC2*-overexpression upregulated ATP-binding cassette B4 (ABCB4), which is a root-localised auxin efflux transporter, and auxin-amido synthetase GH3.4, GH3.6, and GH3.17 in *A. thaliana* leaves [31]. However, no change in transcript levels of CK biosynthetic genes was found in *A. thaliana* leaves overexpressing *ADC2* [84]. Moreover, the expression levels of CK dehydrogenase (CKX) that cleaves the CK side chain, producing aldehydes and adenine derivatives, remained unaffected in *ADC2*-overexpressing *A. thaliana* plants, suggesting that Put does not appear to affect CK biosynthesis or signaling [84]. However, it seems that CKs favor Put biosynthesis and inhibit Spd and Spm accumulation in etiolated cucumber cotyledons since treatment with kinetin increased PAO activity, decreased SAMDC activity, along with a decrease in Spd levels, and increased Put content [85]. Other studies performed on cucumber cotyledon to determine the possible relationship between CK and PAs revealed that kinetin application to excised cotyledons caused a significant increase in the activity of ADC, which was accompanied by an increase in Put content and a decrease in Spd and Spm levels [86]. However, the inhibition of Put biosynthesis with D-Arg did not affect CK-induced expansion of cotyledons and applied alone; Put had no significant effect on growth. In contrast, PAs and ethylene have antagonistic roles. For example, Put treatment decreased ethylene production [87], and this phytohormone was shown to be an effective inhibitor of ADC and SAMDC [88]. The effects of Put on plant growth and development are summarized in Figure 2.

## 3. Tolerance Responses to Abiotic Stresses

The general effect of Put has long been known; it not only participates in plant growth and developmental processes but also contributes to the tolerance to different abiotic stresses such as salinity, drought, high temperatures, and cold. The main described mechanisms are associated with scavenging free radicals, regulating ABA levels, preventing lipid peroxidation, maintaining cellular pH and ionic balance, and regulating cationic channels, among others [89]. These different mechanisms could be induced simultaneously or separately in order to reduce membrane damage, promote cell growth, or enhance cell survival under stress constraints [90]. Furthermore, as previously described, the exogenous application of Put to normal and stressed conditions or the use of transgenic plants overexpressing genes responsible for PAs biosynthesis as well as the loss of function mutants are the main tools used to identify PA-dependent stress responses. Thus, in recent decades, the research community has investigated the effect of PAs when plants are exposed to sole or combined stressors. For example, *ADC1* is mostly activated by cold [91], and *ADC2* expression is induced under drought, salt, and mechanical injury stresses [91,92,93,94,95]. Thus, the effect of the Put pathway on plants affected by the major abiotic stresses (salinity, drought, cold, heat and cold), which negatively influence plant performance [96], will be addressed.

One of the most detrimental environmental stressors is salinity, which produces an osmotic imbalance, ionic toxicity, and oxidative stress [97]. Concerning the PAs role, Zapata et al. [98] described that salt stress might lead to increased (Spd+Spm)/Put ratio, and specifically, the plants more tolerant to salt stress displayed a reduction in Put accumulation. In fact, the increase of Put levels that produces a reduction in this ratio could even cause plant damage. In addition, alfalfa plants under salinity stress exhibited an increase in Spm content at the expense of the decrease of Put and Spd, indicating a deviation of PAs metabolism towards the synthesis of increasing polycationic forms [99]. Similar results were reported by Krishnamurthy and Bhagwat [100] and Santa Cruz et al. [101], showing that rice and tomato tolerant cultivars accumulated Spd and Spm, whereas salt-sensitive cultivars accumulated Put. However, the effect seems to be dependent on the type of stress, time of exposure, and plant species [102]. Leaves of *Lupinus luteus* seedlings accumulated Put and Spd in response to salt stress [103]. Moreover, Quinet et al. [104] showed that the exogenous application of Put diminished Na^+^ levels in roots of a salt-sensitive rice cultivar, leading to an increase in Put and conjugated PAs biosynthesis. In *A. thaliana*, the role of Put in the alleviation of the deleterious effects of salt stress seems to be produced by the induction of *AtADC2* [105]. Support is provided by evidence that the characterization of the *adc2-1* mutant under salt stress is more sensitive than the wild type, but they were recovered by the addition of exogenous Put. Likewise, *Camellia sinensis* cultivars displayed an alleviation of salinity stress through the reduction in the antioxidant enzymes superoxide dismutase (SOD), peroxidase (POD), and catalase (CAT) and the positive effect on photosynthetic efficiency when treated with Put [106]. In line with this, Ekinci et al. [107] indicated that the exogenous application of Put improved plant height, number of leaves, stem diameter, FW, tissue electrical conductivity, and the activity of CAT of pepper seedlings grown under salt stress conditions. Put application also had an effect on reducing CAT and POD activities and increasing carotenoid levels in *Psidium guajava* under salt stress, which seems to be related to the involvement of Put in plant growth via photosynthesis [108]. A study in *Cucumis sativus* showed that Put alleviated stress not only by inducing thylakoid membrane lipid peroxidation by increasing unsaturated fatty acid content but also upregulating ATPase facilitating the Na^+^ efflux [109]. Altogether, this suggests that Put application enhances the ability of PSII-repairing reaction centers and regulates protein expression at transcriptional levels by increasing endogenous PAs content in thylakoid membranes, thereby stabilizing the photosynthetic apparatus under salinity [109]. In addition, Put seems to be actively involved in the glycolytic pathway and the Krebs cycle by means of the inhibition of carbohydrates over-accumulation in leaves and improving the energy formation to face up the salinity damage [110]. Focusing on the biosynthesis pathways of Put, it should be noted that exogenous application of Put could increase endogenous PA levels through ADC pathways in cucumber seedlings under salt stress [111]. *pRD29A*:oat *ADC* transgenic lines showed a smaller reduction in shoot biomass and a slight enhancement in root growth and were healthier than the wild type in response to stress [112]. In addition, *ADC* overexpression led to an osmotic adjustment via the release of proline (Pro) and the increase in K^+^ uptake by roots with a concomitant reduction of Na^+^ accumulation, maintaining Na^+^/K^+^ ratio [112]. A decrease in Na^+^/K^+^ and Na^+^/Ca^2+^ ratios were found in roots when exogenous Put was added, which was associated with root growth promotion [113]. Leaves of *Prosopis strombulifera* treated with Na_2_SO_4_ displayed low Put levels accompanied by the reduction of shoot growth [114]. Nevertheless, roots treated with Na_2_SO_4_ showed an increase in the Put content which was correlated with the formation of adventitious and lateral roots [115,116]. Moreover, an upregulation in *ADC* and *ODC* was observed when NO was exogenously applied in order to synthetize more Put and, in turn, Spd and Spm by NO-induced expression of S-adenosyl methionine decarboxylase (*SAMDC*), *SPDS,* and *SPMS* in tomato plants under sodic alkaline stress [117]. In addition to this statement, Recalde et al. [118] showed that *CuAO8 A. thaliana* mutants reported lower NO levels in response to salt stress, which was probably due to a higher arginase activity preventing Arg availability from affecting the production of NO via nitric oxide synthase-like pathway. On the other hand, García-Jiménez et al. [119] reported that hyposaline shock led to an accumulation in free Put, Spd, and Spm due to a decline in the transglutaminase (TGase) activity, which was accompanied by an increase in the L-Arg pathway.

Another natural abiotic factor that is expected to become more prevalent in terms of frequency and intensity as a result of climate change is drought, being one of the main abiotic factors affecting crop productivity in the Mediterranean regions [120]. Drought causes a reduction in plant growth due to modifications in photosynthesis, nutrient metabolism, ion uptake and translocation, respiration, and carbohydrates metabolism [121]. Focusing these effects on PAs metabolism, it should be noted that Put application by spraying increased leaf area, height, leaf area, and grain yield of wheat plants owing to the increase in chlorophyll, water status, and the content of Pro, amino acids, and soluble sugars [122]. Zhu et al. [123] showed that foliar Put application to lettuce subjected to drought conditions triggered a reduction in stomatal density, keeping chloroplast structure and cell turgor. Similarly, Shallan et al. [124] described that Put application as pretreatment in cotton plants improved root to shoot ratio, leaf area, number and setting of bolls, seed cotton yield, total soluble sugars, pigments content, Pro content, total free amino acids, total phenols, total soluble proteins, total antioxidant capacity, and antioxidant enzyme activities. Put treatment also reduces the sensitivity of *Medicago sativa* plants to PEG-induced drought stress by reducing the activity of the hydrolytic enzymes and increasing the polysaccharide, protein and photosynthetic pigment contents, and photosynthetic activity [125]. Put has the ability to improve anatomical features, retaining chlorophyll concentrations and accumulating total soluble phenolic compounds in *Thymus vulgaris* plants, which leads to improved oil yield under drought conditions [126]. Following this line, Put application promoted drought tolerance in Cabernet Sauvignon seedlings by increasing net photosynthesis rate, the activities of SOD, POD and CAT, levels of ascorbic acid and glutathione, and PA pool [127], as well as in safflower plants via increasing antioxidant enzyme activities, anthocyanin and soluble protein contents, and decreasing lipid peroxidation, electrolyte leakage, and H_2_O_2_ contents [128]. Put-sprayed sugar beet plants suffered less oxidative stress than those not treated, as indicated by lower H_2_O_2_ and MDA accumulation, which is mostly due to the enhanced antioxidant enzyme activities that regulated ROS homeostasis [129]. NO is a key messenger in plant responses, and the interplay with PAs metabolism under drought conditions was studied in transgenic barley plants overexpressing the non-symbiotic hemoglobin gene *HvHb1*, which oxidizes NO to NO_3_^−^ [130]. These plants displayed an increase in Put and Spd, which were correlated with amino acid precursors of PAs and with the expression of specific PA biosynthesis genes. In addition, exogenous Put treatment led to an enhancement of the phospholipase D activity, an enzyme that plays a role in drought stress mitigation at early stages [131]. Foliar application of Put (especially at 150 ppm) prevented the degradation of leaf proteins and chlorophyll and decreased Pro by reducing water deficit stress [132]. Alcázar et al. [133] showed that *A. thaliana ADC2* overexpressor lines displayed a reduction in transpiration rate and stomatal conductance, which indicates that one of the mechanisms involved in the drought tolerance is associated with a reduction of water loss by transpiration. Thus, Put accumulation seems to be an ABA-dependent metabolic response under drought stress, which was revealed due to the impaired Put levels in ABA-deficient and ABA-insensitive *A. thaliana* mutants subjected to this stress [95]. These results resemble those reported in other studies, suggesting that Put acts directly as a protective compound through the activation of the antioxidant machinery to scavenge ROS and prevent lipid peroxidation, thereby contributing to the maintenance of membrane integrity, as well as the accumulation of secondary metabolites under water deficit [134]. Nevertheless, Put application not only improves the drought resistance at vegetative developmental stages but also as seed priming pretreatment leading to an improvement of seed germination, vigor, and enhanced tolerance in maize plants [135]. The correlation between Put content and the degree of resistance to drought seems to be dependent on plant species since a correlation was found in *A. thaliana,* but no correlation was found in rice plants [133,136]. Nonetheless, *ADC* expression was much more induced than *ODC* in response to drought, but the fold change in *ODC1* transcript abundance was linearly correlated with the drought tolerance of the cultivars [136]. Similar results were found by Espasandin et al. [112], showing a direct correlation between *ADC* expression levels and drought tolerance in *Lotus tenuis* transgenic plants overexpressing the oat *ADC* gene. These plants also revealed an upregulation of the 9-cis-epoxycarotenoid dioxygenase (*NCED*) gene under drought conditions, indicating an interrelationship between Put and ABA. It is also worth mentioning that drought stress led to an increase in Put content and PAO and ODC activities in the roots of tolerant maize cultivars compared to susceptible ones [137]. It should be noted that exogenous H_2_S increased the total free PAs, together with an upregulation of *SoADC*, *SoCPA,* and *SoODC* genes in *Spinacia oleracea* seedlings under drought stress, suggesting that H_2_S enhanced tolerance by regulating PA biosynthesis [138].

Plant performance is also negatively affected by soil flooding, an environmental factor that occurs seasonally. However, climate change models predict an increase in the frequency of flooding events worldwide [139]. Flooding stress produces a reduction in relative water content, chlorophyll content, stomatal conductance, and photosynthetic efficiency [140]. The accumulation of Put induced plasma membrane H(+)-ATPase activity in flooded roots, helping to the maintenance of plant cell homeostasis and nutrient uptake [141]. When Carrizo Citrange (*Citrus sinensis* × *Poncirus trifoliata*) and Volkameriana (*Citrus volkameriana*) rootstocks were treated with Put, an improvement of growth and physiological parameters and a reduction of oxidative damage were observed under flooding conditions [142]. Similar results were observed in Put-treated welsh onion plants resulting in the alleviation of flooding stress by the reduction of relative water content, plant growth, chlorophyll fluorescence, and ROS, and the upgrade in the antioxidant system [143].

The progressive increase in the mean global air temperature associated with climate change may adversely affect plant growth and productivity [144,145]. Photosynthesis is one of the most sensitive physiological processes to warmer temperatures. PAs protect plants from high temperatures affecting photosynthesis through the maintenance of thermostability of thylakoid membranes [146,147]. In wheat plants exposed to heat stress during 4 h and 8 h, Hassanein et al. [148] reported a reduction in the growth parameters yield components, as well as in the level of Put, total PAs, and amino acids. In contrast, pretreatment with Arg or Put before exposure to heat stress led to improved tolerance via enhancing the content of Put, Spd, total PAs, and amino acids and decreasing ethylene and NH_4_^+^ content. In accordance with these results, Mostafa et al. [149] showed that foliar application of Arg or Put (1.25 and 2.5 mM, respectively) improved growth and all yield parameters of late sowing wheat plants under heat stress. However, the foliar application of Put combined with the supply of the same proportion of NO_3_^−^/NH_4_^+^ in the nutrient solution alleviated the negative effects of the thermal stress, increasing the content of various sugars, total phenolic compounds, PAs, and the activity of antioxidant enzymes in cauliflower plants (*Brassica oleracea*) [150]. It was also reported that the treatment with PAs 2 h before the application of heat stress at 45 °C for 2 h, as well as the combined treatment of PAs with DFMO, increased the recovery growth of root and hypocotyls in soybean seedlings, whereas when DFMO was supplied alone plants were more vulnerable to heat shock [151]. Additionally, PAs were found to be involved in the reduction of electrolyte leakage and MDA levels, reflecting their role in protecting membrane integrity. These findings also suggest that PAs may replace Ca^2+^ and contribute to the maintenance of membrane integrity by binding to membrane phospholipids under the studied conditions. In line with the previous observation, plants treated with combined heat shock and Arg or Put at varying concentrations showed a significant increase of the antioxidant machinery, in particular SOD and CAT activities [152]. Moreover, PAs can also influence heat-shock protein (HSP) synthesis, which plays an important role in the integrity of cell membranes under high-temperature stress [153]. Melatonin pretreatment increased heat tolerance of tomato seedlings by enhancing the antioxidant defense mechanism and reprogramming the PAs metabolic and NO biosynthesis pathways, which allowed us to scavenge the excess of ROS and improve cellular membrane stability. Melatonin induced respiratory burst oxidase (*RBOH*), heat shock transcription factors A2 (*HsfA2*), heat shock protein 90 (*HSP90*), and delta 1-pyrroline-5-carboxylate synthetase (*P5CS*) gene expression, aiding ROS detoxification [154]. Furthermore, the exogenous application of Put increased *HSP17* transcript levels, and the impact was more marked in thermotolerant cultivars than in the susceptible ones [155]. Moreover, microarray analysis showed that *ADC* overexpression up- and down-regulated several genes involved in hormone and signaling pathways, such as the genes encoding transcription factors belonging to the APETALA2/ethylene-responsive factor domain family [156]. Altogether, this reflects the duality of Put by its direct role and the indirect participation in the acclimation processes [44].

As outlined above, temperature is one of the main abiotic factors that limits plant performance, distribution, productivity, and survival [157]. In this review, the role of PAs under high-temperature conditions were already described, but the role of low temperatures should also be addressed. Put levels increased within 12 h after exposure to 4 °C in *A. thaliana* plants [158]. The exogenous application of Put improved tolerance to chilling in tomato plants by reducing H_2_O_2_ and MDA levels and modulating the antioxidant machinery [159]. The involvement of ABA was also described in Put-induced tolerance to chilling stress in tomato seedlings [160]. ABA treatment could alleviate the electrolyte leakage induced by D-Arg. Hummel et al. [161] showed that ADC seemed to play a more important role than the ODC pathway in PAs biosynthesis in *P. antiscorbutica* seedlings at low temperatures. A positive correlation was observed between agmatine content and primary root growth rate, whereas no correlation was found with Put, or they were even negatively correlated. In this regard, the expression of *BrrADC2.2* followed a cumulative pattern in accordance with Put levels in Tibetan turnip (*Brassica rapa*) under freezing conditions [162]. It was found that BrrICE1.1 (Inducer of CBF Expression 1) could directly interact with the BrrADC2.2 promoter, activating BrrADC2.2 to stimulate the accumulation of Put levels. Furthermore, knock-out mutants for *A. thaliana ADC1* showed increased sensitivity to freezing, but the exogenous application of Put reversed that phenotype [158]. It should be noted that Put also induced seed priming, improving germination, seedling growth, and alleviation to low temperatures [163]. Concerning fruit quality, Abbasi et al. [164] showed that Put application reduced the rate of fruit softening, fruit weight losses, total soluble solids, titratability, ascorbic acid content, and fading of skin colour during storage in peach fruit during low- temperature storage, regardless of the doses of Put applied, or the time of application in *Antirrhinum Majus*. Finally, Put may also play a role in plant tolerance to other abiotic stresses, including heavy metal toxicity or UV radiations, which can lead to yield losses and hamper food security as recently reviewed [25,165]. Several studies demonstrated an accumulation of different PAs accompanied by an increase of ADC when plants were exposed to heavy metals [2,166], i.e., Cd effects on Put accumulation by the activation of ADC was previously described in *Phaseolus vulgaris* seedlings by means of the application of DFMA and DFMO [167]. Concerning UV radiations, which could affect DNA or damage the physiological processes, it should be mentioned that tobacco callus subjected to UV-C radiation displayed higher concentrations of Put in the upper layers, especially in the first 6 h of exposure [168]. Put also protected hulless barley from damage due to UV-B stress via H_2_S- and H_2_O_2_-mediated signaling pathways and their interaction increased plant tolerance by maintaining redox homeostasis and enhancing the accumulation of UV-absorbing molecules [169].

In general, exogenous Put application or the use of *ADC/ODC*-overexpressed plants displayed enhanced tolerance to abiotic stresses and improved the parameters associated with growth, photosynthetic capacity, and antioxidant activity. Several examples of *ADC* and *ODC* transgenic plants subjected to different abiotic stresses are shown in Table 1.

## 4. Resistance Responses to Biotic Stresses

PAs not only play important roles in abiotic stress but also in the regulation of plant defense responses against pathogens [177]. In many cases, the effect of PAs in plant defense is explained by the basis of the production of H_2_O_2_ through PA oxidation [178]. Put is biosynthesized in plants by ADC and ODC routes but Put content is also conditioned by the catabolism activity of copper amine oxidase (CuAO), which oxidizes the primary amino groups of Put, generating the corresponding aldehyde, H_2_O_2_, and NH_4_^+^ [179,180]. ROS production through PA oxidation is proposed to underlie many of the PA functions, including defense signaling [181]. Thus, in *A. thaliana*, AMINE OXIDASE1 (ATAO1/CuAOβ) and CuAO γ1, α3, and ζ exhibited high affinity for Put and loss-of-function mutations in *CuAO* compromise basal defenses. Treatment with Put (500 μM) or the avirulent SAR-inducing *Pseudomonas syringae* pv. *tomato* DC3000 AvrRpm1 triggered systemic resistance in *CuAO* mutants (*atao1-3*, *cuao1-3*, *cuao2-1,* and *cuao3-1*), suggesting that the different CuAOs additively contribute to Put-triggered systemic responses [182,183]. However, plants not only activate PAs catabolism pathways against pathogens but the activation of the biosynthetic routes are also observed. There is evidence that the ADC, ODC, SAMDC, and DAO activities were upregulated in a TMV resistant tobacco line during viral infection, while no changes were observed in the susceptible line [184]. Similarly, barley (*Hordeum vulgare*) infected with the powdery mildew fungus *Blumeria graminis* f. sp. hordei displayed an increase in Put, Spd, and Spm concentrations in infected leaves, while the ADC and ODC activities were also induced [9]. In maize, tumor formation during the interaction with the biotrophic pathogenic fungus *Ustilago maydis* increased the levels of free and conjugated Put [185]. Moreover, the ODC seems to be responsible for the PA increase in wheat leaves infected by *Puccinia graminis* f. sp. *tritici* [186]. In this case, a reduction of the ADC activity was found, whereas the ODC activity significantly increased in the pustules.

The accumulation of Put and Spm was related to an increase in the resistance of tobacco plants infected with *Sclerotinia sclerotiorum* [187]. In addition, the overexpression of *arginase 2* in *A. thaliana* produced an increase in the Put and Pro contents, which is involved in a major resistance to *Botrytis cinerea* infection [188]. The *ADC* gene induction in transgenic eggplants (*Solanum melongena*) with a constitutive promoter of cauliflower mosaic virus-induced resistance against fungal wilt disease caused by *Fusarium oxysporium* and different abiotic stresses [172]. In *A. thaliana*, the expression of both *ADC* isoforms was reported to increase during the hypersensitive response triggered by the avirulent cucumber mosaic virus [189]. Despite having evidence of the protective effect of the Put during defense, signaling pathways underlying PA functions were not elucidated. Kim et al. [14] showed that Put is involved in MAPK cascades regulation producing an increase in *AtADC2* expression during *A. thaliana* response to *P. syringae* infection. In addition, a reduction in Put content in the *adc2* mutant led to an increase in susceptibility to *P. syringae* inoculation. Liu et al. [190] also showed that Put was accumulated in response to flg22, a well-characterized pathogen-associated molecular pattern (PAMP). Through the analysis of *adc1* and *adc2* loss-of-function mutants deficient in Put biosynthesis, it was found that the *ADC2* isoform was the major contributor to Put biosynthesis triggered by flg22. Moreover, exogenous Put application induced defense responses, such as callose deposition and up-regulation of several PAMP triggered immunity (PTI) marker genes. Put could be involved in amplifying PTI responses through ROS production, enhancing disease resistance against bacterial pathogens. In addition, Liu et al. [183] reported that defense signaling triggered by Put partly depends on SA accumulation in *A. thaliana*. Indeed, Put elicits ROS-dependent local SA accumulation and produces a local and systemic reprogramming of genes involved in systemic acquired resistance (SAR). Recently, Rossi et al. [191] demonstrated that Put supplementation reduces plant susceptibility to *P. syringae* in *A. thaliana*, showing an interconnection between SA signaling and plant PA metabolism. This was also supported by the finding that SA treatment led to the upregulation of *ADC1* and *ADC2* genes, whereas the SA treatment of *adc1* and *adc2* loss of function mutants showed that *adc2* had no effect on PA levels. Therefore, ADC2 seems to be the ADC isoform that makes the major contribution to Put accumulation in *A. thaliana* regulated by SA signaling. Avirulent *Xanthomonas campestris* pv. *vesicatoria* was shown to trigger hypersensitive cell death in *Capsicum annuum* via ADC. By binding CaADC1, AvrBsT promoted ROS production, defense gene expression, and cell death [192]. On the contrary, this effector acts as a defense suppressor in tomato plants [193], but no studies about its interaction with tomato ADCs were reported. Recently, tomato *ADC1* and *ADC2* genes were found to be targeted by the *Brg11* effector protein produced by *Ralstonia solanacearum* [194]. *Brg11* induced the production of *ADC* transcripts with higher translational activity than the native mRNA, promoting Put accumulation. However, this perturbation of plant PA metabolism induced by *Brg11* did not seem to affect the infection by *R. solanacearum,* but reduced host susceptibility to *P. syringae*, suggesting that *Brg11* allows *R. solanacearum* to manipulate host defense, obtaining an advantage over other pathogen competitors [194].

Generally, reduced Put levels, in *adc* loss of function mutants and *adc*-silenced lines, result in pathogen susceptibility [183,195]. Sánchez-Rangel et al. [51] generated a transgenic line of *A. thaliana* that silences *ADC1* and *ADC2* genes; this *adc*-silenced line is a non-lethal line with reduced *ADC* gene expression and low Put levels and high levels of ROS. Chávez-Martínez et al. [195] examined the response of this *adc*-silenced line against two pathogens with different lifestyles. This silenced line was more susceptible to *Botrytis cinerea*, and the expression of *PR1* was upregulated, while the jasmonic acid-related genes *LOX3* and *PDF1.2* and *PAD3* involved in camalexin biosynthesis were downregulated. On the other hand, the *ADC*-silenced line increased their resistance to *P. syringae* infection associated with the upregulation of *PR1*, *ZAT1.2*, *WRKY54*, and *WRKY70* genes. The differences in plant defense responses against the two pathogens could be related to the accumulation of ROS previously reported for this *ADC*-silenced line, in which the deregulation of SA- and JA-response genes occurred.

The protective effect of Put was also reported in plants infected by nematodes. *A. thaliana* root infection by the cyst nematode *Heteodera schachtii* increased expression of *ADC1* and *ADC2* genes [196]. The exogenous application of Put can suppress nematode development in tomato plants [197]. Further research is needed to better understand the involvement of Put in plant response to nematode infection.

As previously mentioned, free and conjugated Put levels are enhanced in plant tissues infected by fungi and bacteria [185,198]. In most cases, Put levels in infected tissues are the result of *de novo* biosynthesis [192], although it was also proposed that Put is excreted by the pathogen during plant tissue colonisation [198]. Vilas et al. [198] explained the accumulation of Put in the whole leaf tissues, as well as in the apoplast of tomato plants infected with the bacterial *P. syringae* by the induction of its synthesis in plant cells and also by the excretion of bacteria. The excretion of Put by *P. syringae* was stimulated under virulence inducing conditions, but no activation of bacterial virulence traits or induction of plant invasion were observed after the exogenous addition of Put. However, the possibility that Put could elicit plant defense response cannot be ruled out because the experiment was conducted in plants unchallenged with the pathogen. In some cases, pathogens can modulate plant PA metabolism for their own benefit. In this sense, Stes et al. [199] demonstrated that *Rhodococcus fascians* produces CKs that induce Put accumulation in *A. thaliana* by activating *ADC* expression, increasing symptom development. In addition, the N source can promote an accumulation of Put enhancing resistance to biotic stress. In tomato plants, Fernández-Crespo et al. [200] demonstrated that NH_4_^+^ nutrition confers resistance to *P. syringae* by enhancing H_2_O_2_ accumulation that acts as a signal for activation of systemic acquired acclimation (SAA) mediated by ABA and Put. The high level of the Put precursor Arg hints towards the importance of the glutamate pathway as a key metabolic checkpoint in this pathosystem under NH_4_^+^ nutrition [201]. In addition, Wimalasekera et al. [33] suggested that PA could induce NO production by an unknown pathway. The occurrence of the interplay among PAs, NO, and H_2_O_2_ in stress responses is an interesting phenomenon that requires further investigation.

In plant–pathogen interactions, not only free PAs are involved. PAs are precursors for secondary metabolites and conjugated with phenolic acids which are linked with plant–pathogen defense responses [9,202]. Plant phenolamides, hydroxycinnamic acid amides, or phenylamides identified from the phenylpropanoid derivative pathway are involved in plant defense against bacteria, viruses, fungi, and insects [203,204,205]. Concretely, Put derivatives as caffeoylputrescine increased dramatically in local and systemic tissues of *Nicotiana attenuata* after herbivore attack [206], and p-coumaroylputrescine and feruloylputrescine were strongly accumulated in rice (*Oryza sativa cv. Nipponbare*) leaves subjected to the attack of chewing and sucking herbivores [207]. An accumulation of these phenolamides was also observed in response to *Alternaria brassicicola* challenge in *A. thaliana* rosette leaves [208]. However, further studies are required to identify the mechanisms of these compounds for inducing plant immunity against pathogens.

Several examples of *ADC* and *ODC* transgenic plants subjected to different biotic stresses are shown in Table 2.

Other studies showed that the exogenous application of phytohormones associated with plant defense modified PA metabolism. SA can induce the accumulation of PAs by activating the expression of *ADC* and *ODC* in maize, tobacco, and tomato [209,210,211]. The exogenous application of SA induced PA metabolism in *A. thaliana*, increasing Put levels by ADC activity induction. These changes were *NPR1*-independent and partially dependent on MPK6 activity [191]. In contrast, Liu et al. [183] found that exogenous SA did not affect PA levels in *A. thaliana*. However, this discrepancy can probably be attributed to different experimental conditions used in both studies. The application of the methyl-SA (a SA derivative) to cherry tomato plants also contributed to the accumulation of Put, Spd, and Spm, which was associated with the upregulation of *ADC* and *ODC* genes [209]. The treatment of barley primary leaves with methyl-jasmonate (MeJA) induced the accumulation of free and conjugated Put and Spd, as well as the ADC, ODC, SAMDC, and DAO activities [212]. Similarly, increased PA levels and the activity of enzymes involved both in PA biosynthesis and oxidation were also observed in wheat [213]. MeJA treatment increased the *ADC2* gene expression, while *ADC1* remained unaltered in *A. thaliana*, suggesting a possible different regulatory pathway for both genes [92]. Two *ODC* genes were also induced in tobacco in response to MeJA, but the effect in plant defense is not yet studied [214]. However, in rice, MeJA produced a transient inhibition of *ADC*, *SAMDC,* and *SPDS* gene expression [215]. ABA was also able to induce Put oxidation at the apoplast of *Vicia faba* [216]. This was demonstrated to be important for stomatal closure, an important mechanism to prevent bacterial entry in plants. Nevertheless, more research is necessary to understand the complex connection between SA and ABA metabolism with PAs in plant biotic stress responses.

## 5. Conclusions and Perspectives

This comprehensive review of PAs metabolism is mainly focused on the findings that were made about the involvement of Put in plant growth and development as well as adaptation responses to both biotic and abiotic stresses. There is enough evidence indicating that agriculture will be impaired by global climate change, which could compromise food security in the next future. According to FAO predictions, food production needs to increase 70% more by 2050 for feeding an increasing world population. Future growing conditions will bring the concurrence of different biotic and abiotic stresses. To deal with these upcoming problems and to ensure food security, different approaches to develop stress-tolerant crops will be required. Thus, the role of PAs in the regulation of plant promotion and protection mechanisms might have future implications in agriculture. Although PAs are widely studied across different plant species, further studies are still required to better understand the role of Put and other PAs in the intricate molecular mechanisms underlying plant growth and development and tolerance to abiotic and biotic stresses. Even though most of the reviewed studies examined the effects of exogenous Put, the increase of endogenous Put production by genetic manipulation is becoming of great interest. Thus, metabolic engineering, together with the current development of different high throughput techniques, will be very useful tools to throw light on the complex interactions of PAs with different metabolic pathways, including primary and secondary metabolism, hormones, etc., under different environmental cues. It is also largely unknown how the PAs pathway is regulated at the transcriptional, translational, and post-translational levels. Finally, it should be noted that the use of external applications of these compounds constitutes a good approach to be exploited in agriculture.

## Figures and Tables

**Table 1:** *ADC* and *ODC* transgenic plants showed enhanced tolerance to different abiotic stresses.

Gene	Source	Plant	Enhanced Tolerance to	Reference
***ADC***	*Avena sativa*	*Oryza sativa*	Salinity	[170]
	*Datura stramonium*	*Oryza sativa*	Drought (PEG8000)	[171]
	*Avena sativa*	*Solanum melongena*	Salinity, drought, high temperature and heavy metal	[172]
	*Avena sativa*	*Arabidopsis thaliana*	Dehydration and low temperature	[173]
	*Poncirus trifoliate*	*Arabidopsis thaliana*	Dehydration and drought	[174]
	*Poncirus trifoliate*	*Arabidopsis thaliana*	Osmotic stress, dehydration, drought and low temperature	[175]
	*Avena sativa*	*Lotus tenuis*	Drought	[112]
***ADC1***	*Arabidopsis thaliana*	*Arabidopsis thaliana*	Low temperatures	[158]
***ADC2***	*Arabidopsis thaliana*	*Arabidopsis thaliana*	Drought	[133]
***ODC***	*Mus musculus*	Tobacco	Salinity	[176]

**Table 2:** Increased expression of *ADC* and *ODC* genes in response to biotic stress in different plant species.

Gene	Plant	Biotic Stress	Reference
*ADC*, *ODC*	*Nicotiana tabacum*	*Tobacco Mosaic virus (TMV)*	[184]
*ADC*, *ODC*	*Hordeum vulgare*	*Blumeria graminis* f. sp. *Hordei fungus*	[202]
*ODC*	*Triticum aestivum*	*Puccinia graminis* f. sp. *tritici*	[186]
*ADC*	*Solanum melongena*	*Fusarium oxysporium*	[172]
*ADC*	*Capsicum annuum*	avirulent *Xanthomonas campestris pv versicatoria*	[192]
*ADC*	*Solanum lycopersicum*	*Ralstonia solanacearum*	[174]
*ADC1*, *ADC2*	*Arabidopsis thaliana*	*Heteodera schachtii*	[196]
*ADC1*, *ADC2*	*Arabidopsis thaliana*	Avirulent *Cucumber Mosaic virus*	[189]
*ADC2*	*Arabidopsis thaliana*	*Pseudomonas syringae*	[14]
*Arginase 2*	*Arabidopsis thaliana*	*Botrytis cinerea*	[188]

## Data Availability

Not applicable.
